# Supplementary material for: CA125 Kinetics as a Potential Biomarker for Peritoneal Metastasis Progression following Taxane-Plus-Ramucirumab Administration in Patients with Advanced Gastric Cancer
Source: Cancers (Basel). 2024 Feb 22;16(5):871. doi: 10.3390/cancers16050871 (PMC10930593; doi:10.3390/cancers16050871)
Supplement: Supplementary file 1 [file cancers-16-00871-s001.zip › cancers-2836570-supplementary.pdf]

Supplementary Materials:

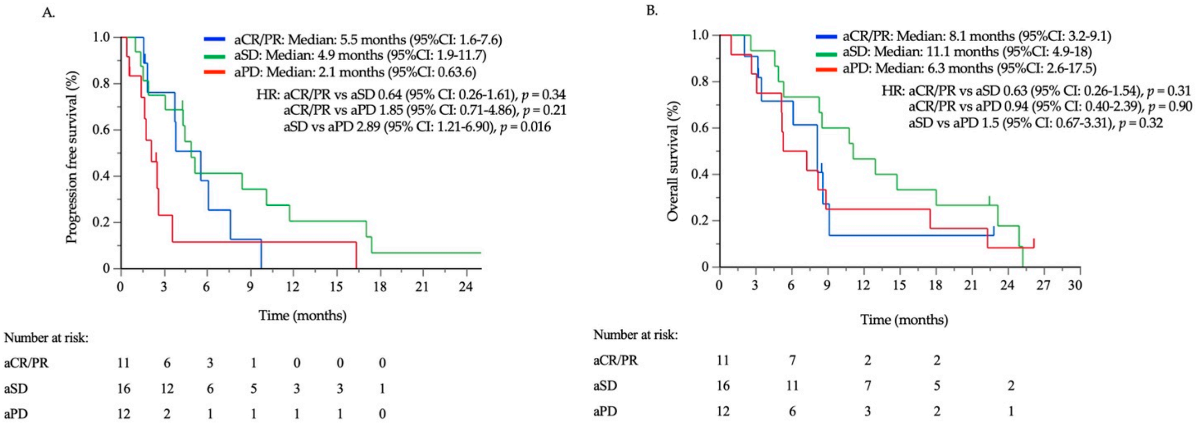

Figure S1. Progression-free survival (A) and overall survival (B) based on ascites response

The survival period based on ascites response did not significantly differ.

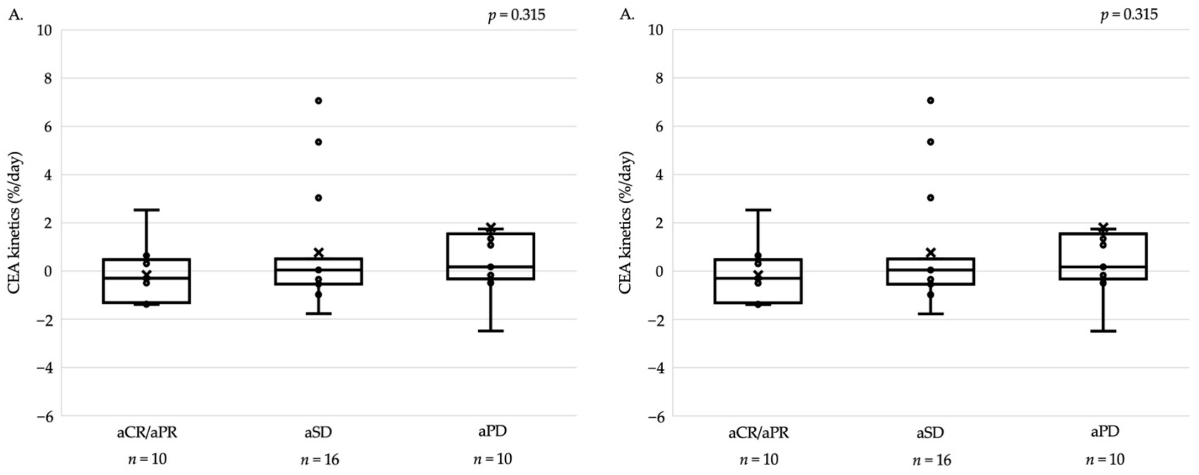

Figure S2. Carcinoembryonic antigen (CEA) and carbohydrate antigen 19–9 (CA19-9) kinetics and ascites response

There was no significant association between CEA (A) or CA 19-9 (B) kinetics and ascites response.

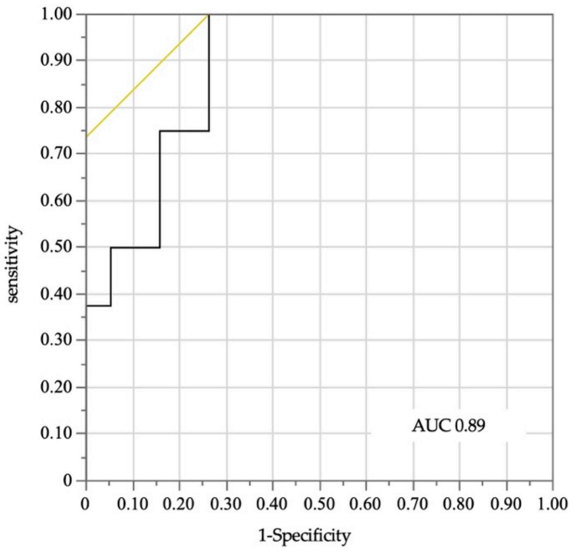

Figure S3. Receiver operating characteristic curve analysis for ascites progression

The optimal cutoff value of carbohydrate antigen 125 (CA125) kinetics to predict ascites progression was 0.0067% per day, resulting in a specificity of 74%, a sensitivity of 100%, and an area under the curve of 0.89.

Table S1. List of sites from which patients were enrolled

Health institution	Principal investigator	Number of patients enrolled
Third Department of Internal Medicine, University of Toyama	Takayuki Ando	36
Department of Internal Medicine, Toyama Red Cross Hospital	Akira Ueda	18
Department of Gastroenterology and Hepatology, Hokkaido University Hospital	Satoshi Yuki	15
Department of Clinical Oncology, University of Miyazaki	Ayumu Hosokawa	4

Table S2. Patient backgrounds in the carbohydrate antigen125-nonincreased and increased groups

		Nonincreased	Increased	*P*-value
Number of patients		24	21	
Age (years)	Median (range)	64 (39–86)	69 (34–79)	0.32
Sex	Male/female	19/5	18/3	0.70
ECOG PS	0	5 (20.8)	6 (28.6)	0.18
	1	15 (62.5)	15 (71.4)	
	≥ 2	4 (16.7)	0 (0)	
Treatment line	2/≥ 3	22/2	17/4	0.40
Metastatic organ (MO)	Liver	6 (0.25)	9 (42.9)	0.23
	Lung	1 (4.2)	3 (14.3)	0.33
	Lymph node	13 (54.2)	9 (42.9)	0.55
	Peritoneum	24 (100)	21 (100)	1
Number of MOs	Median (range)	2 (1–5)	2 (1–4)	0.75

Peritoneal node	Yes	17 (70.8)	13 (61.9)	0.55
	No	7 (29.2)	8 (38.1)	
Ascites	Yes	19 (79.2)	18 (85.7)	0.70
	No	5 (20.8)	3 (14.3)	
	None/mild	16 (66.7)	12 (57.1)	0.55
	Moderate or severe	8 (33.3)	9 (42.9)	
Histopathologic type	Intestinal	8 (33.3)	11 (52.4)	0.24
	Diffuse	16 (66.7)	10 (47.6)	
HER2 status	Positive	3 (12.5)	5 (23.8)	0.45
	Negative	20 (83.3)	16 (76.2)	
	Unknown	1 (4.2)	0 (0)	
Resection of the primary site	Yes	6 (25.0)	7 (33.3)	0.74
	No	18 (75.0)	14 (66.7)	
Duration of the first line*	≥6 months	11 (47.8)	9 (42.9)	0.77
	<6 months	12 (52.2)	12 (57.1)	
Neutrophil count (/μL)	Median (range)	3742 (1575–9416)	3535 (1690–6029)	0.06
Lymphocyte count (/μL)		1185 (370–2490)	1119 (361–3347)	0.83
NLR		2.8 (1.2–12.9)	2.3 (0.6–7.9)	0.27
AST (U/L)		23 (12–138)	27 (12–46)	0.78
ALP (U/L)		323 (167–3844)	310 (138–649)	0.73
LDH (U/L)		219 (132–1811)	215 (167–493)	0.69
CA125 (U/mL)		33.7 (7–507)	24.3 (5.5–317)	0.55
CEA (ng/mL)		3.3 (10.5–648.1)	8.2 (2.3–1549)	0.038
CA19-9 (U/mL)		70 (0.1–7851)	72.8 (0.1–175497)	0.49

Data are presented as n (%) or n/N (%), unless otherwise stated. ECOG, Eastern Cooperative Oncology Group; PS, performance status; HER2, human epidermal growth factor receptor 2; NLR, neutrophil-to-lymphocyte ratio; AST, aspartate aminotransferase; ALP, alkaline phosphatase; LDH, lactate dehydrogenase; CA125, carbohydrate antigen 125; CEA, carcinoembryonic antigen; CA19-9, carbohydrate antigen 19-9. * Data are missing for one patient.
